# Whole-Genome Sequencing Confirms the Coexistence of Different Colonizing Group B *Streptococcus* Isolates Underscored by CRISPR Typing

**DOI:** 10.1128/MRA.01359-19

**Published:** 2020-01-30

**Authors:** Clémence Beauruelle, Maxime Branger, Thierry Cochard, Adeline Pastuszka, Franck Biet, Philippe Lanotte

**Affiliations:** a Université de Tours, INRAE, ISP, Tours, France; b INRAE, Université de Tours, ISP, Nouzilly, France; c CHRU de Tours, Service de Bactériologie-Virologie, Tours, France; University of Rochester School of Medicine and Dentistry

## Abstract

Streptococcus agalactiae is a major pathogen and is the leading cause of neonatal infections in industrialized countries. The diversity of strains isolated from two pregnant women was investigated. Here, we present the draft genome sequences of strains W8A2, W8A6, W10E2, and W10F3, obtained in order to ascertain their phylogenetic affiliation.

## ANNOUNCEMENT

Streptococcus agalactiae, or group B *Streptococcus* (GBS), is a major pathogen in humans and is the leading cause of neonatal infections in industrialized countries (1).

In a recent study, we explored the diversity of GBS in the vaginal carriage population in pregnant women at the time of screening (i.e., at 35 to 37 weeks' gestation by vaginal swabbing according to French clinical guidelines) using a CRISPR typing approach (2). For two women (women 8 and 10), two types of isolates (W8A2 and W8A6 and W10E2 and W10F3, respectively) were identified with similarities among the CRISPR arrays between the two types of isolates.

GBS isolates grew in Todd-Hewitt (TH) broth (BD Biosciences). The genomic DNA was isolated using phenol-chloroform extraction and precipitated with 1 M sodium chloride and 2 volumes of ice-cold 100% ethanol. Whole-genome sequencing and library construction were performed by GenoScreen (Lille, France). Libraries from purified genomic DNA were prepared for Illumina sequencing with a Nextera XT sample prep kit (Illumina, San Diego, USA) according to the supplier’s recommendations. Sequencing was performed using an Illumina HiSeq platform in paired ends of 250 bp. At least 2.8 million reads were obtained for each strain, for a mean coverage of more than 339× for each based on a 2.1-Mb genome size (Table 1).

**TABLE 1:** Sequencing, assembly, and annotation metrics for each strain

Strain	BioSample no.	GenBank accession no.	No. of reads	Avg coverage (×)	No. of contigs	*N*_50_ (kb)	Assembly length (bp)	G+C content (%)	No. of protein CDSs^*a*^
W8A2	SAMN11415932	SSWT00000000	2,851,690	339	50	139,262	2,078,349	35.26	2,059
W8A6	SAMN11415933	SSWU00000000	2,926,588	348	69	99,289	2,086,162	35.29	2,076
W10E2	SAMN11415934	SSWV00000000	2,851,102	339	55	155,056	2,158,522	35.38	2,137
W10F3	SAMN11415935	SSWW00000000	4,173,810	497	55	149,658	2,085,005	35.34	2,097

a CDSs, coding sequences.

A quality check of sequencing data was performed using FastQC v0.11.5 (3), and trimming of reads was done using Sickle v1.33 (4) before assembly using SPAdes v3.11.1 (5). The contigs were annotated using the NCBI Prokaryotic Genome Annotation Pipeline v4.8 (6). All software tools were run using default parameters.

All of the strains were assembled into fewer than 80 contigs (range, 50 to 69) with a total assembly size of 2.1 Mb. About 2,000 coding genes were annotated (range, 2,039 to 2,137). The metrics for each sample are shown in Table 1.

Whole-genome sequencing (WGS) analysis confirmed the presence and structure of CRISPR-Cas genes previously described for these isolates (2). Using PHASTER (7), available for free at http://phaster.ca/, remnant phages were discovered on the 4 genomes, and an intact prophage sequence (79% identity with *Streptococcus* prophage 315.2 [GenBank accession no. NC_004585.1]) was found on W10F3 isolates (contig 4).

In GBS, hemolysin production is encoded by the *cyl* operon, containing 12 genes, including the *cylE* gene (8). Transcriptional regulation of the *cyl* operon is controlled mainly by the two-component system CovS/CovR (*csrS* and *csrR* genes) (9). The *cylE* gene was found strictly conserved in the four different isolates. Even though there was no difference in the *csrS* and *csrR* genes between both types of woman 8 isolates, two mutations were observed in *csrS* genes of isolate W10E2, and one was observed in the W10F3 isolate, which may explain the highly hemolytic activity of W10F3.

Regarding resistance to macrolides, in GBS, erythromycin resistance is commonly due to target modification by an rRNA methyltransferase enzyme encoded by the *ermB* gene (10). An *ermB* gene was found only in isolate W8A6, which presented a high level of resistance to both erythromycin and clindamycin.

**FIG 1:**
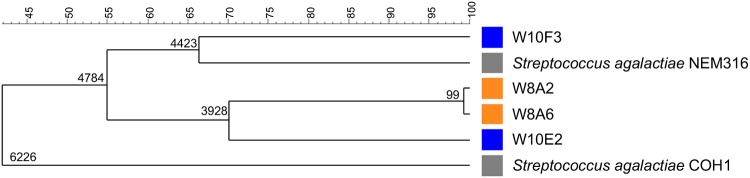
Phylogeny of the clinical isolates of Streptococcus agalactiae with reference genomes, resolved with SNPs. The unweighted pair group method with arithmetic mean (UPGMA) phylogenetic tree was inferred from the 19,460 SNPs detected in the whole-genome sequences of clinical isolates in comparison to the reference strain, Streptococcus agalactiae NEM316. The number of SNPs identified between the strains is indicated along the top.

One of the major outcomes of this study was the degree of relatedness between isolates originating from each woman. The tree based on single nucleotide polymorphism (SNP) analysis (Fig. 1) allowed the exclusion of a phylogenetic affiliation between isolates W10E2 and W10F3, despite them having the same serotype and a common terminal direct repeat and ancestral spacer. The phylogenetic tree with numbering above the branches indicating the similarity coefficient between the strains (Fig. 1) was constructed with concatenation of SNPs using the software BioNumerics v7.6.3 (Applied Maths) with the settings “SNP-based categorical” and “clustering method by UPGMA.” This result confirms the difference in sequence types found (sequence type 4 [ST4] and ST28). Isolates from type W8A2 and type W8A6 were genetically closely related. These results show the *in vivo* coexistence of carriage of 2 types of closely related strains with a probable common ancestor.

### Data availability.

The draft genome sequences of strains W8A2, W8A6, W10E2, and W10F3 have been deposited in DDBJ/ENA/GenBank under the accession no. SSWT00000000 (BioSample no. SAMN11415932), SSWU00000000 (BioSample no. SAMN11415933), SSWV00000000 (BioSample no. SAMN11415934), and SSWW00000000 (BioSample no. SAMN11415935), respectively.
